# Pie‐crusting the medial collateral ligament is a safe and effective technique for improving visualisation and access in arthroscopic meniscal surgery: A systematic review

**DOI:** 10.1002/ksa.70103

**Published:** 2025-10-27

**Authors:** Satyavenkata Kotipalli, Benjamin Blackman, Prushoth Vivekanantha, Marc Daniel Bouchard, Amit Meena, Shahbaz Malik, Darren de SA

**Affiliations:** ^1^ Michael G. DeGroote School of Medicine McMaster University Hamilton Ontario Canada; ^2^ School of Medicine University of Limerick Limerick Ireland; ^3^ Division of Orthopaedic Surgery, Department of Surgery McMaster University Hamilton Ontario Canada; ^4^ Gelenkpunkt – Sports and Joint Surgery FIFA Medical Centre of Excellence Innsbruk Austria; ^5^ Research Unit for Orthopaedic Sports Medicine and Injury Prevention (OSMI) UMIT Tirol – Private University for Health Sciences and Health Technology Innsbruk Austria; ^6^ Department of Orthopaedics and Trauma Shalby Hospital Jaipur Jaipur India; ^7^ Department of Orthopaedic Surgery Worcestershire Acute Hospitals NHS Trust Worcester UK

**Keywords:** arthroscopy, medial collateral ligament, meniscus, percutaneous release, pie‐crust

## Abstract

**Purpose:**

To assess the safety, effectiveness, and postoperative outcomes of medial collateral ligament (MCL) pie‐crusting (PC) in arthroscopic meniscus surgery.

**Methods:**

This systematic review was conducted in accordance with PRISMA guidelines. Three databases (PubMed, EMBASE and MEDLINE) were searched from inception to 21 January 2025, for studies evaluating the use of MCL PC during arthroscopic meniscus or anterior cruciate ligament (ACL) surgery. Data on patient demographics, medial joint space measurements, postoperative instability, patient reported outcome measures (PROMs), and complications were extracted.

**Results:**

Fifteen studies comprising 1009 patients were included, with 723 undergoing PC. PC significantly increased medial joint space width by a mean of 5.7 mm intraoperatively (*p* < 0.05), with no residual laxity reported at mean final follow‐up of 16.5 months. The most common complications of PC were transient medial knee pain (12.7%) and ecchymosis (12.9%). There were five reports of saphenous nerve irritation (0.8%), all resolved by final follow‐up of 31.6 months. In contrast, iatrogenic chondral injury was reported in 9.0% of patients undergoing meniscal repair without PC.

**Conclusion:**

MCL PC is a safe and effective technique to improve visualisation and outcomes in arthroscopic meniscus surgery. It is associated with improved joint access and low complication rates without long‐term instability. While current evidence supports its use, further high‐quality, long‐term comparative studies are needed to validate its safety and efficacy, as this study primarily used retrospective data without long‐term follow‐up.

**Level of Evidence:**

Level IV.

**Abbreviations**
ACL: anterior cruciate ligament
BMI: body mass index
IKDC: International Knee Documentation Committee
KOOS: Knee Injury and Osteoarthritis Outcome Score
MCL: medial collateral ligament
PC: pie‐crusting
PRISMA: Preferred Reporting Items for Systematic Reviews and Meta‐Analyses
RTS: return to sport

## INTRODUCTION

Meniscal tears are a common cause of knee pain, with a prevalence of 12%–14% in the general population [37]. Arthroscopy has allowed these tears to be repaired with minimally invasive surgery, as open meniscus surgery is rarely performed [46]. Despite the advantages of arthroscopy, a considerable disadvantage compared to open surgery is the decreased visibility and difficulty of instrument manipulation through arthroscopic ports in narrow compartments [25]. Specifically, the posteromedial compartment and medial posterior horn have been associated with decreased visualisation and increased instrument breakage [19, 36, 44]. Inadequate visualisation of meniscal tears can lead to inadequate repair, iatrogenic chondral damage and subsequent revisions [9, 50, 52]. Cartilage damage during surgery has short‐term effects, including pain and limited range of motion, and in the long term can cause degenerative joint changes [26, 41]. Therefore, adequate visualisation, especially in the medial compartment, which most commonly becomes osteoarthritic, is crucial in meniscus procedures to optimise outcomes [39].

To overcome this limitation in arthroscopic visualisation, several modifications have been proposed. One common technique to increase medial compartment joint space involves applying a valgus force and externally rotating the knee [1]. However, uncontrolled excess valgus force has been shown to rarely cause iatrogenic medial collateral ligament (MCL) injury and femoral avulsion fractures [31]. A study from 2004 described a technique to increase medial joint space by purposefully puncturing the MCL [2]. This technique, now known as pie‐crusting (PC), involves percutaneous punctures over the MCL with the knee in valgus stress with a needle, allowing for increased medial joint space in a controlled manner [30]. Pie‐crusting mitigates the complications of isolated valgus force, such as severe MCL injury and avulsion fractures. Although this technique will require the MCL to heal post‐operatively, the MCL has a significantly greater healing potential than articular cartilage, which rarely heals after damage [6, 32].

There are several proposed complications with pie‐crusting, with the main concern being medial knee instability from MCL laxity [17, 47]. The most recent reviews assessing pie‐crusting in arthroscopic meniscus surgery were in 2020, consisting of four and six studies, respectively [21, 42]. One study only looked at pie‐crusting the superficial MCL and had average follow‐up time less than one year while the other did not describe PC indications or techniques [18, 38]. Since then, several studies have been published on this topic. This systematic review aims to provide an update on the role of MCL PC in intraoperative visualisation of the medial joint space, post‐operative meniscal outcomes, and potential complications. It is hypothesised that this updated systematic review with more recent literature will provide evidence PC is a safe option for increasing intraoperative joint space for arthroscopic meniscal surgery.

## MATERIALS AND METHODS

This systematic review was conducted in accordance with the Preferred Reporting Items for Systematic Reviews and Meta‐Analyses (PRISMA) [33].

### Search strategy

Three online databases (EMBASE, MEDLINE and PubMed) were searched from inception to 21 January 2025, for studies discussing the use of MCL percutaneous release during arthroscopic meniscus or resection surgery with or without anterior cruciate ligament (ACL) surgery. Search terms included ‘pie‐crusting’, ‘percutaneous release’, ‘medial collateral ligament’, ‘arthroscopy’ and ‘meniscus’ (Supporting Information: Table 1).

**Table 1:** Demographic data including study design, MINORs score, number of patients with and without PC, mean age, number of female patients, mean BMI, follow‐up and lost to follow‐up.

Author (year)	Study design (level of evidence)	MINORS	Number of patients (knees)	Mean age (SD) [range]	Female (*n*) (%)	BMI (SD)	Time to follow‐up (months) (SD)	Lost to follow‐up (*n*, %)
Arıcan et al. [3] (2023)	Retrospective cohort (3)	15/24	PC: 42 Non‐PC: 44	PC: 43 (8.3) Non‐PC: 45 (9.1)	PC: 22 (52.3%) Non‐PC: 19 (43.1%)	PC: 24 (5.3) Non‐PC: 25 (6.2)	PC: 14 (1.3) Non‐PC: 16 (3.1)	0
Arıcan et al. [4] (2021)	Retrospective cohort (3)	10/16	Total: 67 sMCL: 34 dMCL: 33	Total: 48 (3) sMCL: 47 (5) dMCL: 49 (4)	Total: 27 (40.3%) sMCL: 15 (55.5%) dMCL: 12 (445%)	sMCL: 27 (4) dMCL: 24 (3)	sMCL: 15 (3) dMCL: 16 (2)	0
Chung et al. [12] (2017)	Retrospective cohort (3)	16/24	PC = 118 Non‐PC = 20	PC: 57.1 (9.1) Non‐PC: 55.7 (8.2)	PC: 101 (85.6%) Non‐PC: 18 90%)	PC: 26.2 (2.1) Non‐PC: 26.0 (2.3)	PC: 42.4 (19.3) Non‐PC: 37.2 (7.8)	0
Claret et al. [13] (2016)	Retrospective cohort (3)	16/24	PC = 70 Non‐PC = 70	PC: 44 (19–62) Non‐PC: 47 (18–60)	PC: 26 (37.2%) Non‐PC: 22 (31.5%)	NR	Up to 6 months	0
Ercan et al. [15] (2024)	Retrospective cohort (3)	20/24	PC = 32 Non‐PC = 36	PC: 58.9 (3.8) Non‐PC: 59.3 (4.2)	PC: 16 (50%) Non‐PC: 17 (47.2%)	PC: 26.5 (3.9) Non‐PC: 26.2 (4.4)	PC: 34.1 [24–42] Non‐PC: 35.9 [24–49]	NR
Erdem et al. [16] (2021)	Retrospective cohort (3)	9/16	53	29.4 [14–49]	4 (7.5%)	NR	31.6 [24–45]	NR
Fakioglu et al. [17] (2013)	Case series (4)	12/16	18	Median: 43 [22–59]	NR	NR	Median: 8.3 [6–12]	NR
Gabr and Robinson [20] (2024)	Retrospective cohort (3)	19/24	Total: 55 Proximal PC: 34 Distal PC: 21	30 [16–50]	22 (40%)	NR	50.43 [24–84]	NR
Han et al. [24] (2020)	Retrospective cohort (3)	11/16	60	37.0 (5.3) [16–55]	28 (46%)	25.0 (2.1)	21.93 (7.04) [12–36]	NR
Polat et al. [47] (2020)	Prospective cohort (2)	12/16	18	38.5 (9.2) [21–58]	5 (27.8%)	NR	8.9 (2.0) [6–12]	0 (0%)
Moran et al. [42] (2021)	Prospective cohort (2)	12/16	27	55.3 (10.7) [31–77]	20 (47.6%)	NR	1.5	15 (35.7%)
Herber et al. [27] (2024)	Retrospective cohort (3)	19/24	PC: 45 Non‐PC: 52	PC: 55.2 (10.9) Non‐PC: 57.2 (8.5)	PC: 34 (75%) Non‐PC: 40 (77.8%)	NR	24	0 (0%)
Jeon et al. [30] (2018)	Retrospective Cohort (3)	20/24	PC: 64 Non‐PC: 64* *1:1 matching	PC: 40.9 (12.5) Non‐PC: 42.6 (15.8)	PC: 23 (35.9%) Non‐PC: 25 (39.1%)	PC: 24.6 (2.7) Non‐PC: 25.1 (3.5)	24	0 (0%)
Lons et al. [35] (2018)	Retrospective cohort (3)	13/16	40	39 (9) [20–54]	7 (17.5%)	NR	1.5	0 (0%)
Srisuwanporn et al. [53] (2024)	Retrospective cohort (3)	12/16	14	50 (10) [35–63]	10 (71.5%)	26.3 (3.8)	3	0 (0%)

Abbreviations: BMI, body mass index; dMCL, deep medial collateral ligament; MCL, medial collateral ligament; MINORS, Methodological Index for Non‐Randomised Studies; *n*, number; NR, not reported; PC, pie‐crusting; SD, standard deviation; sMCL, superficial medial collateral ligament.

Inclusion criteria included: (1) studies using MCL PC or MCL release, (2) studies performing arthroscopic meniscus surgery with or without concomitant operations such as ACL reconstructions, (3) studies reporting on outcomes of surgery including but not limited to patient reported outcome measures, postoperative laxity, return to sport (RTS) and complications, (4) human studies and (5) studies in English. Exclusion criteria included: (1) systematic reviews and meta‐analyses, (2) book chapters and (3) case reports.

### Study screening

Abstract and title screening was done independently by two authors (S.K. and B.B.) and any disagreement about inclusion was resolved after a consensus was reached by the same two authors. When a consensus could not be reached, a more senior author was consulted. Similarly, during full text review studies were screened by the same two authors and conflicts were resolved in a similar manner.

### Quality assessment

Quality of the studies selected for this systematic review was assessed using the Methodological Index for Non‐Randomised Studies (MINORS) criteria [51]. Based on this criteria, comparative studies could get a maximum score of 24 and were categorised as very low quality (0–6), low quality (7–10), fair quality (11–15), good quality (16–20) and high quality (≥20). Non‐comparative studies could get a maximum score of 16 and were categorised as very low quality (0–4), low quality (5–7), fair quality (8–12) and high quality (≥13) [7]. Furthermore, quality assessment was performed to assess for bias in studies using the 7‐item Risk Of Bias In Non‐randomised Studies of Interventions (ROBINS‐I) for non‐randomised studies [54].

### Assessment of agreement

During title and abstract screening as well as full text review, the inter‐reviewer agreement to include a study in this systematic review was assessed using the kappa (*κ*) statistic. Agreement was categorised as almost perfect agreement (0.91–0.99), considerable agreement (0.71–0.90), high agreement (0.61–0.70), moderate agreement (0.41–0.60), fair agreement (0.21–0.40) or no agreement (≤20) [40].

### Data abstraction

Data was collected and summarised from the screened articles by two authors and compiled using Google Sheets spreadsheet (Google LCC, Mountain View, CA, USA). Demographic data was collected, including the number of patients, mean age, sex, body mass index (BMI), follow‐up time and patients lost to follow‐up. Surgical details collected included indications for pie‐crusting, the technique used and the surgical technique used for meniscus repair. Outcomes related to the percutaneous MCL release included postoperative joint distance and angle, instability, revision and complications. Patient reported outcome measures (PROMs) were also extracted and included the following: the International Knee Documentation Committee (IKDC) scores [29], Lysholm scores [10], Knee Osteoarthritis and Outcome Scores (KOOS) [48], Tegner activity scores [10], Oxford Scores [14], subjective pain and subjective instability.

### Statistical analysis

Results from the studies were reported in a descriptive fashion. Google Sheets (Google LLC) was used to summarise mean, ranges and percentages. Studies which performed subgroup analysis and found statistical significance were reported with *p*‐values included. If multiple studies reported statistical significance about the same parameter, the highest *p*‐value was reported. Statistical significance was defined as *p* < 0.05. Clinical significance following medial meniscus repair and partial meniscectomy was assessed using a Minimal Clinically Important Difference (MCID) for IKDC of 10.9 and 10.6, respectively [23, 38].

## RESULTS

### Literature search

**Figure 1:**
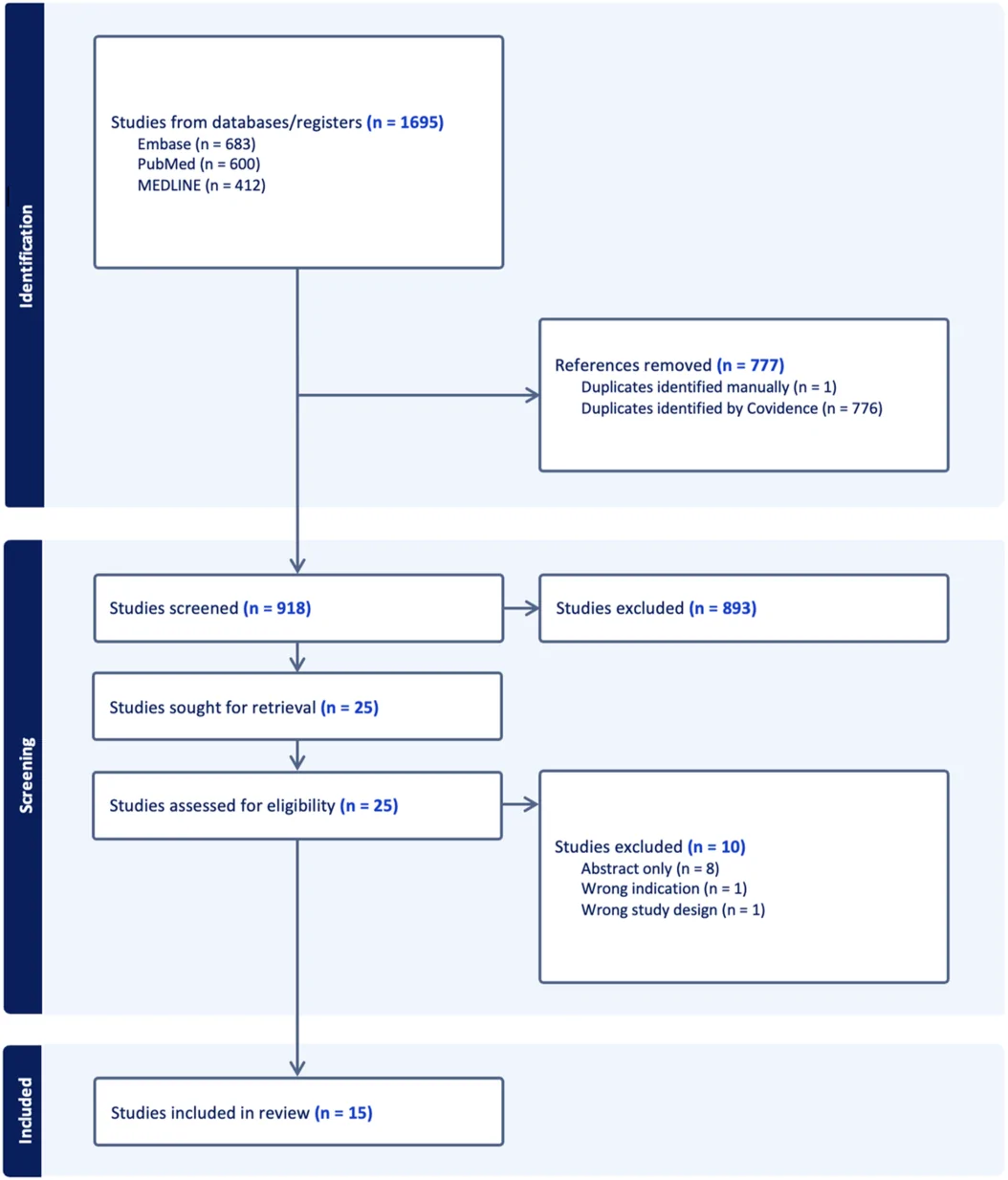
Preferred Reporting Items for Systematic Reviews and Meta‐analyses (PRISMA) flow diagram representing a systematic review on the use of pie‐crusting in arthroscopic knee surgery.

The initial search across PubMed, Embase and MEDLINE yielded 1695 studies, with 777 duplicates identified. A total of 918 studies underwent title and abstract screening, with 25 studies being selected for full‐text review. Overall, 15 studies satisfied eligibility criteria and were selected for final analysis (Figure 1) [3, 4, 12, 13, 15, 16, 17, 20, 24, 27, 30, 35, 42, 47, 53]. There was considerable agreement during both title and abstract screening (*k* = 0.81, 95% confidence interval [CI]: 0.691–0.932) and during full text analysis (*k* = 0.74, 95% CI: 0.467–1.000).

### Study quality

Of the 15 studies included in this systematic review, one was a case series (level of evidence IV), 12 were retrospective comparative (level of evidence III), and two were prospective comparative (level of evidence II). The mean MINORS score was 74.4% for the comparative studies and 71.9% for the case series. All studies were either of fair, good or high quality. Twelve studies were found to have low to moderate risk of bias, while three studies were found to have at least one element of severe risk of bias as per ROBINS‐I. Full details of risk of bias can be found in Supporting Information: Table 2.

### Patient demographics

Across the 15 studies included in this systematic review, there were a total of 1009 patients. Of these patients, 723 underwent PC with meniscal procedure while 286 had meniscus procedures without PC. One study consisting of 53 patients had 31 patients undergo concomitant ACL reconstruction [16]. Fourteen studies consisting of 991 patients reported the mean age of patients undergoing procedures with and without PC to be 45.0 years (range: 29.4–58.9) and 49.7 (range: 42.6–59.3), respectively [3, 4, 12, 13, 15, 16, 20, 24, 27, 30, 35, 42, 47, 53]. One study consisting of 18 patients who underwent PC reported a median age of 43 years (range: 22–59) [17]. There were 345 (48.9%) and 141 (49.3%) female patients who did and did not undergo PC, respectively. Seven studies consisting of 397 patients who underwent PC and 164 patients who did not undergo PC reported an average BMI of 25.4 and 25.4, respectively [4, 6, 12, 15, 22, 38, 48]. Thirteen studies consisting of 635 patients who underwent PC reported average follow‐up times of 25.8 months (range: 1.5–50.4) [4, 6, 12, 15, 16, 19, 22, 25, 28, 32, 38, 42, 48]. One study consisting of 27 patients reported that 15 patients were lost to follow‐up [42], with the remaining studies either reporting no patients lost to follow‐up or not reporting at all. Full details about patient demographics can be found in Table 1.

### Surgical details

Indications for PC were reported in 14 studies consisting of 668 patients [3, 4, 12, 13, 15, 16, 17, 24, 27, 30, 35, 42, 47, 53]. All 14 studies reported insufficient medial joint space for adequate visualisation or instrumentation as the primary indication for PC. Four studies consisting of 259 patients reported there must have been a posterior medial meniscus lesion along with a narrow medial compartment for PC to be performed [12, 13, 16, 47]. Narrow joint space was most commonly defined as less than 5 mm of medial joint width or was decided subjectively by the surgeon. Full details about PC indications can be found in Table 2.

**Table 2:** Surgical details including indications for pie‐crusting, technique used for pie‐crusting and the meniscal procedure done.

Author	Indication for Pie‐crusting	Pie‐crusting technique	Meniscus surgical technique
Arıcan et al. [3]	Medial joint width <5 mm.	Limb in full extension, valgus force of 150 N applied and several deep skin punctures with 18 G needle performed to release sMCL. Punctures made along a line marked from 1.5 to 2 cm posterior inferior to medial epicondyle and 2.5–3 cm anterior‐inferior to adductor tubercle.	Medial meniscus repair: 42 (100%)
Arıcan et al. [4]	Medial joint width <5 mm.	Several skin punctures with 18 G needle under 150 N valgus force, confirmed with popping sound and increased medial joint space. Surgeon preference for deep vs superficial MCL release. Superficial MCL released at proximal tibial insertion, deep MCL and meniscocapsular ligament release posterior to anterior.	Medial meniscus repair: 67 (100%)
Chung et al. [12]	Medial meniscus posterior root repair AND narrow medial joint space, poor visual field and insufficient working space. Avoided in medial insufficient patients.	Periosteum elevator directed toward the distal attachment of sMCL and released via subperiosteal stripping in 2 directions being distal and posteromedial. dMCL preserved and proximal attachment of sMCL preserved.	Pullout medial meniscus repair technique using simple stitch of modified Mason–Allen stitch: 118 (100%)
Claret et al. [13]	Medial meniscus posterior horn lesion AND Medial joint width <5 mm.	Under valgus force, repeated 20 G needle punctures to the posterior and medial MCL through a single cutaneous puncture, at the level of the posterior horn of the medial meniscus. Confirmed under direct visualisation.	Partial Medial meniscectomy: 70 (100%)
Ercan et al. [15]	Insufficient joint space under 150 N valgus force at 30° flexion.	Under valgus force, an 18 G needle was used through a single percutaneous puncture at the posterior third of medial meniscus then the deep MCL was partially released from the tibial side via 4–5 punctures in the posteromedial region. Release confirmed by sudden pop and increased joint space. Confirmed under direct visualisation.	Medial partial meniscectomy: 32 (100%)
Erdem et al. [16]	Difficult cases (posterior horn tear, large longitudinal midsubstance tear, tears with extension into midsubstance, bucket handle tears) AND tight medial joint space	15°–20° knee flexion and gentle valgus stress. 3–5 percutaneous punctures with 18 G needle on MCL from anterior to posterior superior to the medial joint line. Confirmed under direct visualisation.	Medial meniscus repair: 53 (100%) Meniscal suturing without tying, if ACLR was done meniscal stitches were tied over the capsule medially and posteromedially
Fakioglu et al. [17]	Insufficient visualisation of posteromedial structures under 11‐kg valgus stress	With 16 G venous cannula under valgus stress the posterior third of superficial MCL proximal to medial meniscus was punctured. If opening was inadequate, needle was used to release more posteromedial capsuloligamentous structures in horizontal plane until desired medial joint opening was attained, maximum of 4 punctures.	Partial medial meniscectomy: 18 (100%)
Gabr and Robinson [20]	NR	proximal MCL: Under valgus force and 10°–15° of flexion, 18 G needle was used on the posterior proximal fibres of the sMCL, just proximal to the medial meniscus and distal to medial femoral condyle. distal MCL: Sartorius fascia was divided proximal to pes anserinus exposing distal tibial attachment of sMCL, a valgus force was applied and stab incisions were made just proximal to distal tibial attachment of sMCL.	Medial meniscus repair: 36 Medial + lateral meniscus repair: 8 Medial partial meniscectomy: 4 Medial meniscus repair + partial lateral meniscectomy: 4 medial meniscus repair + chondroplasty medial femoral condyle: 1 ramp lesion repair: 2
Han et al. [24]	Narrow medial joint space or posteromedial meniscus not visible	At level of medial joint line under valgus force, multi‐point percutaneous pie‐crusting of posterior MCL with 18 G needle until medial compartment opened.	Meniscoplasty: 40 Suturing: 20
Polat et al. [47]	All knees with posteromedial compartment cases AND inadequate visualisation.	Multiple punctures were performed with an 18 G needle to the posteromedial part of the MCL, while the controlled valgus force was maintained visualising the medial compartment with the arthroscope. Done at the level of the joint.	Partial medial meniscectomy: 13 (72.2%) Suturing: 5 (27.8%)
Moran et al. [42]	Insufficient medial compartment access, determined intraoperatively.	1.5 cm posterior and distal to the medial epicondyle, constant valgus force applied to the knee, an 18 G spinal needle was used to fenestrate the superficial MCL with rapid anterior‐to‐posterior translation until an audible pop was heard, confirmed under direct visualisation.	Partial medial meniscectomy: 27 (100%)
Herber et al. [27]	Posteromedial meniscus root or capsular attachment superior to posterior horn not visualised.	Under valgus stress, an 18 G needle was used to create several percutaneous fenestrations in the proximal MCL. Complete MCL release was avoided.	Medial meniscal root repair: 45 (100%)
Jeon et al. [30]	Medial joint space too narrow for proper inspection of the entire meniscus lesion OR instrumentation could not be inserted.	Posterior third of the MCL just above the medial meniscus was targeted with a 19 G intravenous catheterisation needle. Posteromedial ligamentocapsular complex was pierced 2–4 times, until medial gap is wide enough.	PC: Meniscal repair: 34 (53.1%) Meniscectomy: 25 (39.1%) Root repair: 5 (7.8%) Non‐PC: Meniscal repair: 27 (42.2%) Meniscectomy: 31 (48.4%) Root repair: 6 (9.4%)
Lons et al. [35]	Tight medial joint space.	Intramuscular needle, submeniscally, adjacent to the medial meniscus, at the posterior two‐thirds junction of the compartment, under valgus force.	Meniscectomy: 33 (82.5%) Repair: 7 (17.5%)
Srisuwanporn et al. [53]	Medial joint space too narrow for instrumentation and medial meniscus and posterior root cannot be visualised.	18 G needle was carefully punctured at the “magic point” on Thammasat line 2.8 cm distal to the adductor tubercle, 1.8 cm distal to the medial epicondyle, and 1.2 cm above the medial joint line.	Meniscal repair: 14 (100%)

Abbreviations: ACLR, anterior cruciate ligament reconstruction; cm, centimetre; dMCL, deep medial collateral ligament; G, gauge; MCL, medial collateral ligament; mm, millimetre; N, Newton; PC, pie‐crusting; sMCL, superficial medial collateral ligament.

All studies reported on the specific technique used for PC, with 14 studies consisting of 605 patients reporting using a technique involving valgus stress of the knee followed by percutaneous punctures of the MCL using a needle to increase medial joint space [3, 4, 12, 13, 15, 16, 17, 20, 24, 27, 30, 35, 42, 47, 53]. The most common needle gauge used was 18 G, reported in 10 studies consisting of 413 patients [3, 4, 13, 14, 17, 20, 23, 26, 31, 38, 42, 48]. The location of where the MCL was released varied widely across all studies. The most common location was slightly superior to the medial meniscus, reported in four studies consisting of 190 patients [16, 17, 20, 30]. Release adjacent to the medial meniscus was reported in three studies consisting of 142 patients [13, 15, 35] and release at the level of the joint line was reported in two studies consisting of 78 [24, 47]. Full details, including the site and number of the puncture wounds, amount of valgus stress and size of needle used can be found in Table 2. One study consisting of 118 patients reported using a periosteal elevator and releasing the distal attachment of the superficial MCL via subperiosteal stripping, starting inferior from the pes anserinus attachment and aiming distally towards the superficial MCL attachment [12].

Meniscal procedures performed after PC were reported in all 15 studies [3, 4, 12, 13, 15, 16, 17, 20, 24, 27, 30, 35, 42, 47, 53]. Of the patients who underwent PC, the most common meniscus procedures were medial meniscal repairs performed in 461 patients (63.7%) with 50 of those patients having medial meniscus root repairs (6.9%). Other procedures included partial medial meniscectomy performed in 164 patients (22.7%), unspecified medial meniscectomy performed in 58 patients (8.0%), and meniscoplasty performed in 40 patients (5.5%). Full details of all meniscal procedures can be found in Table 2.

### Visualisation and mechanical alignment

The average preoperative medial joint space width reported in 11 studies consisting of 462 patients was 5.5 mm (range: 1.9–9.35), measured with either arthroscopic probe or radiographs [3, 4, 15, 16, 27, 30, 35, 42, 47, 53]. The average medial joint space width after PC reported in six studies consisting of 239 patients was 10.4 mm (range: 7.82–12.5), with an average increase in medial joint space width of 5.7 mm [3, 4, 15, 16, 42, 47]. Five studies consisting of 186 patients reported a statistically significant increase in joint space width following PC (*p* < 0.05) [3, 4, 15, 42, 47]. Eight studies consisting of 346 patients reported medial joint space width of 5.7 mm (range: 4.5–10.4) at an average final follow up time of 16.5 months (range: 1.5–34.1). All studies reported no statistically significant difference from the preoperative joint space width at the time of final follow‐up [3, 4, 15, 24, 30, 35, 42, 53].

Four studies consisting of 181 patients reported on valgus laxity angle preoperatively and at the time of the final follow‐up [3, 4, 15, 35]. The average angle preoperatively was 4.1 degrees (range: 1.1–5.6) and the average angle at the time of final follow‐up was 4.4° (range: 1.9–5.7). No significant difference was found in all four studies between the angle preoperatively and at an average final follow up of 15.3 months (range: 1.5–34.1). Three studies consisting of 141 patients reported on the joint angle intraoperatively after PC and found an increase of 3.0° (range: 2.5–3.4) [3, 4, 15]. All three studies reported this as a statistically significant increase in joint angle (*p* < 0.05) [4, 6, 15]. Full details can be found in Table 3.

**Table 3:** Joint space width and angle for PC patients including pre‐operative, intraoperative and at time of final follow‐up.

Author	Joint space width (mm)	Joint angle
Arıcan et al. [3]	Preop: 3.2 [2.5–4.4] Intraoperative after PC: 11.4 [8.6–12.3], statistically significant Postop 1 month: 10.1 [8.2–10.9] Postop 6 month: 9.2 [5.8–10.2] Postop 12 month: 6.2 [3.2–7.8] n.s.	Preop = 5.2 [4.6–5.8] Intraoperative after PC = 7.7 [6.5–9.9], statistically significant Postop 1 month = 7.5 [6.0–9.6] Postop 6 month = 6.5 [6.0–7.9] Postop 12 month = 5.6 [4.9–6.6] n.s.
Arıcan et al. [4]	sMCL Preop: 4.5 (3.6–4.9) Intraoperative after PC: 10.4 (7.9–12.1), statistically significant 1 month postop: 8.4 (7.8–11.2) 6 months postop: 6.8 (6.1–9) 12 months postop: 4.3 (4–6.3), n.s. dMCL Preop: 4.3 (3.5–4.8) Intraoperative after PC: 11.2 (8.3–12.5) 1 month postop: 9.2 (8.8–10.2) 6 months postop: 7.5 (6.8–9.3) 12 months postop: 4.8 (4.4–6.7), n.s.	sMCL Preop: 4.1 (3.3–5.2) Intraoperative after PC = 7.5 (6.6–9.1), statistically significant 1 month postop: 6.7 (5.3–8.2) 6 months postop: 5.8 (5.2–9.1) 12 months postop: 4.3 (3–5.5), n.s. dMCL Preop: 4.7 (4.4–5.6) Intraoperative after PC: 8.2 (6.4–9.3) 1 month postop: 7.4 (5.9–8.6) 6 months postop: 6.2 (5.6–8.2) 12 months postop = 4.8 (4.6–5.9), n.s.
Chung et al. [12]	NR	NR
Claret et al. [13]	NR	NR
Ercan et al. [15]	Preop: 5.8 (1.4) Postop: 10.5 (1.6), *p* < 0.05 6 month postop: 8.2 (1.1) 12 month postop: 6.1 (1.3) 24 month postop: 5.9 (1.2)	Preop: 5.6 (1.5) Postop: 8.2 (1.2), *p* < 0.05 6 month postop: 6.7 (1.4) 12 month postop: 5.9 (1.7) 24 month postop: 5.7 (1.9)
Erdem et al. [16]	Preop: 4.61 (0.5) Postop: 7.82 (0.85)	NR
Fakioglu et al. [17]	Preoperative median: 7.1 [3.7–9.6] 1 week postop median: 9.1 [6.2–11.3], *p* < 0.0001 3 month postop median: 8.0 [5.3–10.1] 6 month postop median = 7.2 [3.9–9.8, n.s]	NR
Gabr and Robinson [20]	NR	NR
Han et al. [24]	Preop: 5.9 (0.8) [4–7.5] 1 week postop: 9.2 (1.1) [7–11] *p* < 0.01 3 months postop: 6.1 (0.9) [4.2–7.6] p > 0.05	NR
Polat et al. [47]	Preop: 5.1 (0.9) Valgus force: 7.8 (1.4) After PC: 12.5 (2.1), *p* < 0.05 compared with preop and Valgus force	NR
Moran et al. [42]	Preop: 5.95 (1.32) After PC: 11.09 (1.74) *p* = 0.00 6 weeks postop: 5.85 (0.99) *p* = 0.474	NR
Herber et al. [27]	PC: Preop: 1.9 (0.8) Non‐PC: Preop: 2.0 (0.7)	NR
Jeon et al. [30]	PC: Preop: 8.2 (1.8) Final: 7.9 (2.2) n.s.	NR
Lons et al. [35]	Preop: 9.35 [6.1–13.3] Final: 10.4 [6.6–14.4]	Pre: 1.1 [0–5] Final: 1.9 [0–6]
Srisuwanporn et al. [53]	Preop: 7.42 ± (1.16) Final: 7.47 (1.15) *p* = 0.914	NR

Abbreviations: dMCL, deep medial collateral ligament; MCL, medial collateral ligament; NR, not reported; n.s., not significant; PC, pie‐crusting; sMCL, superficial medial collateral ligament.

### Complications

Thirteen studies consisting of 604 patients who underwent PC and 222 patients who did not undergo PC reported on complications [3, 4, 12, 13, 15, 16, 17, 24, 27, 35, 42, 47, 53]. The most common complications among patients who underwent PC were transient postoperative pain and ecchymosis at the medial knee reported in 77 (12.7%) and 78 (12.9%), respectively. One study found five reports of saphenous nerve injury (0.8%) diagnosed based on patient symptoms, which were resolved by the time of final follow‐up 31.6 months [16]. In terms of rehabilitation, six studies consisting of 292 patients described bracing post‐operatively to protect the MCL along with limiting flexion [12, 17, 24, 30, 47, 53], and an additional four studies also used bracing postoperatively, but did not report it was specifically for MCL protection [3, 16, 20, 27]. There were no reports of residual MCL laxity or instability at the time of final follow up across all studies.

Among patients who did not undergo PC, the most common complication was iatrogenic chondral injury reported in 20 patients (9.0%) during the procedure and this was found to be statistically higher in non‐PC patients compared to PC patients in two studies (*p* = 0.01, *p* < 0.05) [3, 15]. No incidence of chondral injury was reported in all 15 studies for PC patients. One study consisting of 45 PC patients and 52 non‐PC patients found a statistically significant increase in recurrent meniscal tears among patients who did not undergo PC compared to those who did (*p* = 0.028) [27]. Full details can be found in Table 4.

**Table 4:** Complications of patients with and without PC.

Author (year)	Complications
Arıcan et al. [3] (2023)	Non‐PC: 9 iatrogenic chondral injury (20.5%), *p* = 0.01
Arıcan et al. [4] (2021)	None
Chung et al. [12] (2017)	PC: Some patients had residual valgus laxity up to 1 year
Claret et al. [13] (2016)	PC: 28 reports mild pain lasting 15 days in MCL (23.7%) 48 reports of superficial light ecchymosis over MCL, resolved (40.7%)
Ercan et al. [15] (2024)	Non‐PC: 11 iatrogenic chondral injury, not seen in release group (15.7%), *p* < 0.05 PC: 20 reports of mild pain at medial needle tract for 2 weeks (62.5%) 12 reports of superficial ecchymosis, resolved (37.5%)
Erdem et al. [16] (2021)	PC: 5 patients had saphenous nerve injuries, resolved by final follow‐up (9.4%). 1 patient had recurrent meniscal tear after 2 years, required partial meniscectomy (1.9%).
Fakioglu et al. [17] (2013)	PC: All patients reported mild pain and superficial ecchymosis at the medial needle tract for 15 days (100%). All patients had less than 5 mm opening with valgus stress at 30 degrees flexion which recovered on average of 3.5 weeks (100%).
Gabr and Robinsion [20] (2024)	NR
Han et al. [24] (2020)	Abnormal signal: 5 (25%)
Polat et al. [47] (2020)	PC: 4 reports of medial side pain post‐op (22.2%)
Moran et al. [42] (2021)	PC: 7 reports of medial side pain post‐op (26%)
Herber et al. [27] (2024)	PC: 2 reports of stiffness (3.9%) 1 reported infection (1.9%) 2 conversions to TKA (3.9%) Non‐PC: 2 reports of stiffness (4.4%) 4 recurrent tears (8.9%), *p* = 0.028 2 reports of infection (4.4%) 4 conversions to TKA (8.9%)
Jeon et al. [30] (2018)	NR
Lons et al. [35] (2018)	0 (0%)
Srisuwanporn et al. [53] (2024)	0 (0%)

Abbreviations: MCL, medial collateral ligament; NR, not reported; PC, pie crusting; TKA, total knee arthroplasty.

### PROMs

Eight studies consisting of 443 patients undergoing PC reported on IKDC with a mean score of 77.4 (range: 50.9–85.1) at mean follow up time of 30.3 months (range: 1.5–50.4) [3, 12, 15, 20, 24, 27, 30, 42]. MCID percentages were significantly higher at the time of final follow up compared to preoperative scores for all patients who underwent PC in all eight studies, for both medical meniscus repair and meniscectomies. Seven studies consisting of 379 patients reported the final IKDC score to be significantly higher than the preoperative IKDC score (*p* < 0.05) [3, 12, 15, 20, 24, 27, 42]. Nine studies consisting of 502 patients undergoing PC reported a mean Lysholm score of 86.5 (range: 80.1–93.0) at mean follow up time of 25.6 months (range: 6–42.4) [3, 12, 13, 15, 16, 24, 27, 30, 47]. Seven studies consisting of 385 patients found the Lysholm score at the time of final follow‐up to be significantly higher than the preoperative Lysholm score (*p* < 0.05) [3, 12, 13, 15, 17, 24, 47]. Two studies consisting of 77 patients who underwent PC found a significantly higher IKDC score and Lysholm score at mean follow‐up of 28.2 (range: 24–34.1) compared to patients who did not undergo PC (*p* < 0.5) [15, 27]. Full details about PROMs can be found in Table 5.

**Table 5:** PROMs including IKDC, Lysholm, Tegner and VAS of patients with and without PC.

Author	IKDC	Lysholm	Tegner	VAS
Arıcan et al. [3]	PC: Preop: 47.41 (18.82) 1 month postop: 62.97 (11.00), significantly higher than non release group (*p* = 0.01) 6 month postop: 71.54 (13.36) 1 year postop: 70.30 (18.66), significantly higher than preop Non‐PC: Preop: 46.95 (17.60) 1 month postop: 55.2 (13.67) 6 month postop: 62.94 (12.85) 1 year postop: 69.58 (12.82), significantly higher than preop	PC: Preop: 48.25 (20.36) 1 month postop: 66.5 (17.47), significantly higher than non release group (*p* = 0.02) 6 month postop: 82.5 (16.22) 12 months postop: 89.15 (9.2), significantly higher than preop Non‐PC: Preop: 45 (25.35) 1 month postop: 55.8 (17.80) 6 month postop: 69.30 (16.72) 1 year postop: 85 (13.22), significantly higher than preop	PC: ‐ Preop = 4.3 (2.41) ‐ 1 month postop = 3.9 (1.47), significantly higher than Non‐PC group (*p* = 0.01) ‐ 6 month postop = 5.2 (1.94) ‐ 1 year postop = 5.5 (1.76), significantly higher than preop Non‐PC: ‐ Preop = 4.95 (2.06) ‐ 1 month postop = 2.75 (1.68) ‐ 6 month postop = 4.15 (1.50) ‐ 1 year postop = 5.9 (1.71), significantly higher than preop	NR
Arıcan et al. [4]	NR	NR	NR	NR
Chung et al. [12]	sMCL preop: 40.1 (7.7) sMCL postop: 73.6 (11.2) *p* < 0.001	sMCL preop: 52.4 (9.1) sMCL postop: 84.4 (12.1) *p* < 0.001	NR	NR
Claret et al. [13]	NR	PC: Preop: 60 (9) 2 months postop: 73 (4), *p* < 0.001 from preop 6 months postop: 92 (4), *p* < 0.001 from preop Non‐PC: Preop: 54 (11) 2 months postop: 70 (4), *p* < 0.001 from preop 6 months postop: 92 (5), *p* < 0.001 from preop Between groups Preop, *p* = 0.001 2 months postop, *p* = 0.019 6 months postop, 0.095	PC: ‐ Preoperative = 3 [2–4] ‐ 2 months = 3 [2–5], *p* < 0.001 from preop ‐ 6 months = 4 [2–5], *p* < 0.001 from preop Non‐PC: ‐ Preoperative = 2 [1–4] ‐ 2 months = 3 [2–4], *p* = 0.002 from preop ‐ 6 months = 3 [2–4], *p* < 0.001 from preop Between groups ‐ Preoperative, *p* = 0.037 ‐ 2 months, *p* = 0.818 ‐ 6 months = 0.099	PC VAS in Activity: ‐ Preoperative = 7 [5–8] ‐ 2 months = 3 [1–5], *p* < 0.001 from preop ‐ 6 months = 1 [0–3], *p* < 0.001 from preop PC VAS at Rest: ‐ Preoperative = 2 [1–4] ‐ 2 months = 1 [0–2], *p* < 0.001 from preop ‐ 6 months = 0 [0–1], *p* < 0.001 from preop Non‐PC VAS in activity: ‐ Preoperative = 6 [5–8] ‐ 2 months = 3 [1–5], *p* < 0.001 from preop ‐ 6 months = 1 [0–3], *p* < 0.001 from preop Non‐PC VAS at rest: ‐ Preoperative = 2 [1–4] ‐ 2 months = 1 [0–2], *p* < 0.001 from preop ‐ 6 months = 1 [0–1], *p* < 0.001 from preop VAS in activity between groups: ‐ Preoperative, *p* = 0.308 ‐ 2 months, *p* = 0.535 ‐ 6 months, *p* = 0.003 VAS at rest between groups: ‐ preoperative, *p* = 0.363 ‐ 2 months, *p* = 0.077 ‐ 6 months = 0.097
Ercan et al. [15]	PC: Preop: 46.7 (13.2) 24 months Postop: 82.2 (15.3) *p* < 0.05 Non‐PC: Preop: 46.4 (10.5) 24 months Postop: 75.4 (12.3) *p* < 0.05 Between groups preop, *p* = n.s. 24 months postop, *p* < 0.05	PC: Preop: 50.1 (6.3) 24 months postop: 86 (7.1) *p* < 0.05 Non‐PC: Preop: 48.4 (6.8) 24 months Postop: 78 (7.3) *p* < 0.05 Between groups preoperative, *p* = n.s. 24 months postop, *p* < 0.05	PC: ‐ Preoperative = 4 [1–5] ‐ 24 months ostoperative = 6 [4–9], *p* < 0.05 Non‐PC: ‐ Preoperative = 4 [1–6] ‐ 24 months Postoperative = 5 [3–8], p < 0.05 Between groups ‐ preoperative, *p* = n.s. ‐ 24 months postoperative, *p* < 0.05	PC: ‐ Preoperative = 5.5 (0.6) ‐ Postoperative = 1.1 (0.5), *p* < 0.05 Non‐PC: ‐ Preoperative = 5.6 (0.7) ‐ Postoperative = 1.9 (0.6), *p* < 0.05 Between groups ‐ preoperative, *p* = n.s. ‐ 24 months postoperative, *p* < 0.05
Erdem et al. [16]	NR	6 months postop: 93 [67–100]	NR	6 months postop = 0.8 [0–4]
Fakioglu et al. [17]	NR	Preop Median: 42 [24–64] Final Median: 94 [88‐100] *p* = 0.0002	NR	NR
Gabr and Robinson [20]	PC: Preop: 44 [32–48] Final: 84 [70–95] *p* < 0.05 Non‐PC: Preop: 58 [44–67] Final: 90 [82–97] *p* < 0.01	NR	PC: ‐ Preoperative = 2 [1–3] ‐ Postoperative = 6 [5–7] Non‐PC: ‐ Preoperative = 3 [2.5–4] ‐ Postoperative = 6 [5–8]	EQ. 5D‐VAS PC ‐ Preoperative = 75 [50–85] ‐ Postoperative = 89 [80–92] EQ. 5D‐VAS Non‐PC ‐ Preoperative = 70 [60–84] ‐ Postoperative = 85 [79–93]
Han et al. [24]	Preop: 51.42 (4.02) Final: 82.17 (4.64) *p* = 0.00	Preop: 48.17 (4.22) Final: 80.08 (3.71) *p* = 0.00	Pre: 3.20 (0.68) Final: 5.48 (0.59) *p* = 0.00	Pre: 5.57 (0.69) Final: 1.80 (0.51) *p* = 0.00
Polat et al. [47]	NR	Preop: 59.3 (7.9) Final: 85.7 (4.7) *p* = 0.00	Pre: 2.5 (0.5) Final: 4.2 (0.4) *p* = 0.00	NR
Moran et al. [42]	Pre: 39.1 (12.7) Final: 50.9 (16.3) *p* = 0.002	NR	NR	NR
Herber et al. [27]	PC: Preop: 44.2 (9.4) 24 month postop: 85.1 (8.2) Non‐PC: Preop: 41.7 (9.1) 24 month postop: 78.7 (11.5) *p* = 0.002	PC: Preop: 46.9 (8.4) 24 month postop: 84.1 (9.6) Non‐PC: Preop: 44.6 (8.4) 24 month postop: 79.0 (9.1) *p* = 0.01	NR	NR
Jeon et al. [30]	PC: Preop: 42.0 (19.5) 24 month postop: 82.4 (19.3) Non‐PC: Preop: 46.6 (19.2) 24 month postop: 81.3 (20.1) n.s.	PC: Preop: 50.7 (24.9) 24 month postop: 85.1 (17.2) Non‐PC: Preop: 57.0 (26.0) 24 month postop: 83.9 (20.4) n.s.	NR	PC: Preop: 3.5 24 month postop: 1.1 Non‐PC: Preop: 3.9 24 month postop: 1.2
Lons et al. [35]	NR	NR	NR	NR
Srisuwanporn et al. [53]	NR	NR	NR	NR

Abbreviations: IKDC, International Knee Documentation Committee; NR, not reported; PC, pie‐crusting; sMCL, superficial medial collateral ligament; VAS, visual analogue scale.

## DISCUSSION

The primary finding of this review is that pie‐crusting is a safe and effective means of accessing the medial joint compartment in arthroscopic knee surgery, with no residual knee laxity or nerve injury. Although there was variation in where PC was done along the MCL, the majority of studies in this review released the MCL just superior to the medial meniscus, at the level of the meniscus or at the level of the joint line. Meniscal procedures with PC resulted in improved clinical outcomes and PROMs compared to preoperative scores, with two studies finding significantly higher PROMs in PC patients compared to patients without PC. The MCID for meniscal procedures ranges from 10.3 to 12.3 for the Lysholm score [34, 55] and from 9.9 to 10.9 for the IKDC subjective score [34, 38]. The studies that showed statistically significant improvements in these PROMs exceeded the respective MCID thresholds, highlighting the clinically significant benefit of the interventions. The patients included in the non‐PC group also exceeded their respective MCID thresholds [3, 13, 15, 20, 27, 30].

Although the MCL is partially released intraoperatively, the induced laxity was transient, with the majority of studies reporting that medial joint space width and valgus stability returned to baseline by the final follow‐up. Complications were minimal, with transient medial sided joint pain and ecchymosis being most common. Less than 1% of patients had saphenous nerve injury, which was resolved by final follow‐up [16]. In this study, the inside‐out repair technique was used, which may have increased the likelihood of transient saphenous nerve injury [11, 16]. Chondral damage, which occurred in 9% of patients in the non‐pie‐crusting group, is particularly concerning due to the limited regenerative capacity of articular cartilage, leading to osteoarthritis [18, 22]. The reduction in meniscus re‐tears implies that PC can lead to a more robust initial repair due to the added visualisation and ease of access and instrumentation manoeuvreing PC provides. The repair success rate reduces the need for revision and associated joint morbidity. This study also found that pie‐crusting is effective in facilitating meniscal repair alongside ACL reconstruction. One study [28] found that patients undergoing meniscal repair with ACLR had higher rates of chondral damage compared to those undergoing ACLR alone. However, another study included in our review [16] found that patients undergoing PC during concomitant ACLR and meniscal surgery did not incur chondral damage, suggesting a potential protective effect of improved visualisation through pie‐crusting.

The majority of studies had similar indications for pie crusting, opting for the technique when there was insufficient medial joint space for adequate visualisation or instrumentation. Most performed PC by applying a valgus stress prior to puncturing the MCL with a needle [45], whereas one study used a periosteal elevator to release the superficial MCL [12]. While some authors favour an inside‐out technique [5], the most commonly performed PC techniques in this review were percutaneous outside‐in approaches, primarily with an 18‐gauge needle aiming at the posterior third of the superficial MCL. This technique has been shown to be safe, easily reproducible, and effective [8, 30, 42, 47]. Although the study employing periosteal stripping reported no subjective instability, clinical examination revealed persistent valgus instability in 6.8% (*n* = 8) patients at final follow‐up [12], suggesting that an outside‐in approach may offer superior stability outcomes. Differences in specific PC techniques (Table 2) may influence the amount of joint space widening, risk for chondral or nerve injury, and the potential for residual MCL laxity.

These findings are similar to those reported in previous systematic reviews on PC. One study [21] looked at 192 patients across four studies and found no complications. Mild postoperative pain at the medial needle tract site lasting up to 15 days was reported in 52% of patients. This contrasts with our review, which noted only 13% of patients reporting postoperative medial pain and ecchymosis. Similar to our findings, no patients in their review experienced subjective instability. Lysholm scores ranged from 85 to 94, whereas scores in our review ranged from 80.1 to 93.0, with a mean of 86.5. A 2023 systematic review assessing pie‐crusting for varus knees in total knee arthroplasty reported no perioperative complications in the pie‐crusting group [43]. Although this involved a different patient population than in our review, these findings further support the overall safety of the pie‐crusting technique in knee surgery. Compared to earlier reviews, this review included more recent studies and provided a direct comparison between PC and non‐PC cohorts undergoing meniscal surgery. These additions advance clinical practice by highlighting that PC is a safe and effective technique in arthroscopic meniscal surgery without long‐term adverse outcomes.

This review is not without limitations. First, the average follow‐up time in the included studies was relatively short, with a mean of just over two years, limiting the ability to assess long‐term complications such as progressive joint degeneration. While no studies included in this review reported ongoing valgus laxity, the biomechanical implications of releasing the MCL and inducing coronal laxity may alter the load distribution in the medial compartment and increase risk of arthritis [49, 56]. Long‐term studies assessing prevalence of osteoarthritis following PC are needed to better quantify the risk. Although the majority of studies had the same indication and overall technique for PC, there was considerable heterogeneity in the specific techniques used. For example, some studies referenced a ‘magic point’ for the percutaneous release of the MCL with specific landmarks for needle insertion. In contrast, other studies described their technique more broadly, using spinal needles to target general areas such as the posteromedial region of the MCL. The differences in the exact location of puncture sites, number of needle insertions, amount of valgus stress, and instrumentation could all influence the degree of intraoperative medial joint space widening in addition to how well the MCL recovers post‐operatively. However, regardless of technique, all studies assessing medial stability at follow‐up reported a return to baseline laxity. Another important limitation is that there were only two prospective comparative studies that allowed for direct comparisons between pie‐crusting and non‐pie‐crusting cohorts [38, 42]. The remaining studies were non‐comparative and retrospective, limiting the strength of conclusions. Future research should focus on high‐quality, long‐term comparative studies with standardised pie‐crusting protocols and well‐defined endpoints, including specific patient subgroups such as athletes involved in cutting or contact sports. While there were fewer re‐tears in the comparative studies in the short‐term follow‐up, it would be worth confirming whether this difference remains long‐term. Additionally, evaluating the incidence of medial compartment osteoarthritis or other medial‐sided injuries in pie‐crusting versus non‐pie‐crusting groups would be valuable in determining whether this technique influences long‐term joint health. Further research is also needed to identify the optimal anatomical point for pie‐crusting and to develop evidence‐based rehabilitation protocols tailored to MCL recovery after percutaneous release.

## CONCLUSION

Pie‐crusting appears to improve visualisation in medial meniscus surgery with no consistent evidence of clinical instability, but long‐term outcomes and high quality comparative data remain limited.

## AUTHOR CONTRIBUTIONS

**Satyavenkata Kotipalli**: Screening; data extraction; writing; editing. **Benjamin Blackman**: Screening; data extraction; writing; editing. **Prushoth Vivekanantha**: Writing; editing; idea conception. **Marc Bouchard**: Writing; editing. **Amit Meena**: Writing; editing. **Shahbaz Malik**: Writing; editing. **Darren de SA**: Writing; editing; idea conception.

## CONFLICT OF INTEREST STATEMENT

The authors declare no conflicts of interest.

## ETHICS STATEMENT

There are no relevant ethical disclosures pertaining to research involving human participants and/or animals, and informed consent was not necessary to develop this manuscript.

## Supporting information

Supp_Table.

## Data Availability

Data may be made available upon reasonable request at bwb531@gmail.com.
