# Supplementary material for: Pie‐crusting the medial collateral ligament is a safe and effective technique for improving visualisation and access in arthroscopic meniscal surgery: A systematic review
Source: Knee Surg Sports Traumatol Arthrosc. 2025 Oct 27;34(9):3298–313. doi: 10.1002/ksa.70103 (PMC13502447; doi:10.1002/ksa.70103)
Supplement: Supplementary file 1 [file KSA-34-3298-s001.docx]

**SUPPLEMENTARY DIGITAL MATERIAL**

Supplementary Table 1: Search terms

n = number of studies

| Pie-crusting OR Ligament pie-crusting OR Pie crust OR percutaneous release OR release AND (medial collateral ligament OR MCL OR medial) AND (ACL OR meniscus OR arthroscopic OR arthroscopy | Pie-crusting OR Ligament pie-crusting OR Pie crust OR percutaneous release OR release) AND (medial collateral ligament OR MCL OR medial) AND (ACL OR meniscus OR arthroscopic OR arthroscopy | Pie-crusting OR Ligament pie-crusting OR Pie crust OR percutaneous release OR release) AND (medial collateral ligament OR MCL OR medial) AND (ACL OR meniscus OR arthroscopic OR arthroscopy |
| --- | --- | --- |
| Pubmed (n = 600) | EMBASE (n = 683) | MEDLINE (n = 412) |

Supplementary Table 2: ROBINS-I

| Study | Bias of confounding | Bias in classification of interventions | Bias in selection of participants into the study | Bias due to deviations from intended interventions | Bias due to missins data | Bias in measurment of outcome | Bias in selection of the reported result |
| --- | --- | --- | --- | --- | --- | --- | --- |
| Arıcan (2023) | M | L | L | L | L | L | L |
| Arıcan (2021) | M | L | L | L | L | L | L |
| Chung (2017) | M | L | L | L | M | S | L |
| Claret (2016) | L | L | L | L | L | L | L |
| Ercan (2024) | M | L | L | L | M | M | L |
| Erdem (2021) | M | L | L | L | M | NA | L |
| Fakioglu (2013) | L | L | L | L | M | NA | L |
| Gabr (2024) | M | L | L | L | L | M | L |
| Han (2020) | M | L | L | L | M | NA | L |
| Polat (2020) | S | L | L | L | L | NA | M |
| Moran (2021) | L | L | L | L | S | NA | L |
| Herber (2024) | M | L | L | L | L | L | L |
| Jeon (2018) | M | L | L | L | L | L | L |
| Lons (2018) | S | L | L | L | L | NA | L |
| Srisuwanporn (2024) | M | L | L | L | L | NA | L |

ROBINS-I = Risk Of Bias In Non-randomized Studies of Interventions L = Low risk of bias, M = Moderate risk of bias, S = Severe risk of bias, NA = Not applicable
